# DNALI1 Promotes Neurodegeneration after Traumatic Brain Injury via Inhibition of Autophagosome‐Lysosome Fusion

**DOI:** 10.1002/advs.202306399

**Published:** 2024-02-13

**Authors:** Xulong Ding, Shuqiang Cao, Qing Wang, Bin Du, Kefeng Lu, Shiqian Qi, Ying Cheng, Qing‐zhang Tuo, Weibo Liang, Peng Lei

**Affiliations:** ^1^ Department of Neurology and State Key Laboratory of Biotherapy National Clinical Research Center for Geriatrics West China Hospital Sichuan University Chengdu 610041 China; ^2^ Center of Translational Medicine and Clinical Laboratory The Fourth Affiliated Hospital of Soochow University Medical Center of Soochow University Suzhou Dushu Lake Hospital Suzhou Jiangsu 215123 China; ^3^ Department of Forensic Genetics West China School of Basic Science and Forensic Medicine Sichuan University Chengdu 610041 China

**Keywords:** autophagy, chronic traumatic encephalopathy, DNALI1, neurodegeneration, traumatic brain injury

## Abstract

Traumatic brain injury (TBI) leads to progressive neurodegeneration that may be caused by chronic traumatic encephalopathy (CTE). However, the precise mechanism remains unclear. Herein, the study identifies a crucial protein, axonemal dynein light intermediate polypeptide 1 (DNALI1), and elucidated its potential pathogenic role in post‐TBI neurodegeneration. The *DNALI1* gene is systematically screened through analyses of Aging, Dementia, and TBI studies, confirming its elevated expression both in vitro and in vivo. Moreover, it is observed that altered DNALI1 expression under normal conditions has no discernible effect. However, upon overexpression, DNALI1 inhibits autophagosome‐lysosome fusion, reduces autophagic flux, and exacerbates cell death under pathological conditions. DNALI1 silencing significantly enhances autophagic flux and alleviates neurodegeneration in a CTE model. These findings highlight DNALI1 as a potential key target for preventing TBI‐related neurodegeneration.

## Graphical abstract

Following TBI, ciliogenesis increases under mechanical and oxidative stress, leading to the increase of DNALI1. DNALI1 prevents the clearance of phosphorylated tau by autophagy, inhibiting autophagic flux and contributing to the development of neurodegeneration.

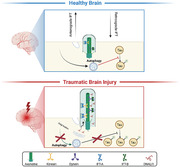

## 1 Introduction

Traumatic brain injury (TBI) refers to the temporary or permanent impairment of brain function caused by external mechanical forces on the head.^[^ ^1^ ^]^ TBI is commonly associated with high morbidity, mortality, disability rates, and other severe adverse outcomes.^[^ ^2^ ^]^ Globally, the annual incidence of TBI varies from 27 to 69 million,^[^ ^3^ ^]^ and it is estimated that approximately half of the world's population will experience one or more TBIs in their lifetime. Long‐term disability affects 43% of patients hospitalized for TBI,^[^ ^4^ ^]^ imposing a significant socioeconomic burden. Despite the prevalence of TBI, current treatments primarily aim to stabilize and alleviate symptoms, often neglecting potential long‐term effects.

TBI stands out as the most significant non‐genetic, non‐age‐related risk factor for dementia.^[^ ^5^ ^]^ The neurodegeneration that follows head injuries or repetitive mild trauma may be caused by chronic traumatic encephalopathy (CTE) alone or in conjunction with Alzheimer's disease (AD). Various severities of TBI can result in different pathological states, and epidemiological studies underscore that 70–90% of TBIs are mild.^[^ ^6^ ^]^ Though not immediately life‐threatening, repeated TBIs induce cumulative effects, ultimately elevating the risk of CTE.^[^ ^7^ ^]^ CTE is a progressive tauopathy with a distinct clinical and neuropathological profile that becomes symptomatic many years after an individual experiences repeated concussive or subconcussive blows to the head.^[^ ^8^ ^]^ The pathology of CTE differs from the clinical and pathological sequelae of severe single‐incident TBI, and it typically involves neurofibrillary tangles (NFTs) accumulating in the superficial gray matter.^[^ ^9^ ^]^ Axon strain results in microtubule rupture and tau release, facilitating its phosphorylation at disease‐related sites, and potentially leading to neurodegeneration.^[^ ^10^ ^]^ Amyloid β pathology mainly manifests in single‐incident TBI, with evident accumulation of amyloid precursor protein in damaged axons and cell bodies in both TBI animals and humans within hours.^[^ ^11^ ^]^ Therefore, the clinical presentation of CTE can be different to AD, emphasizing the importance of investigating the mechanisms underpinning CTE and identifying critical factors with diagnostic, therapeutic, and prognostic value.

In this study, we employed a variety of bioinformatics and statistical approaches, with a particular focus on the hippocampus. By comparing data from all brain regions, we aimed to identify the key pathways or genes involved in post‐TBI neurodegeneration. Subsequently, we validated these findings using in vivo and in vitro TBI models. We conducted targeted gene manipulations using CTE animal model and evaluated the impacts on neurodegeneration and delved into potential underlying mechanisms. These results identify a promising target for preventing CTE.

## 2 Results

### 2.1 *DNALI1* is a Critical Gene to Predict Cognitive Impairment Post‐TBI

**Figure 1:**
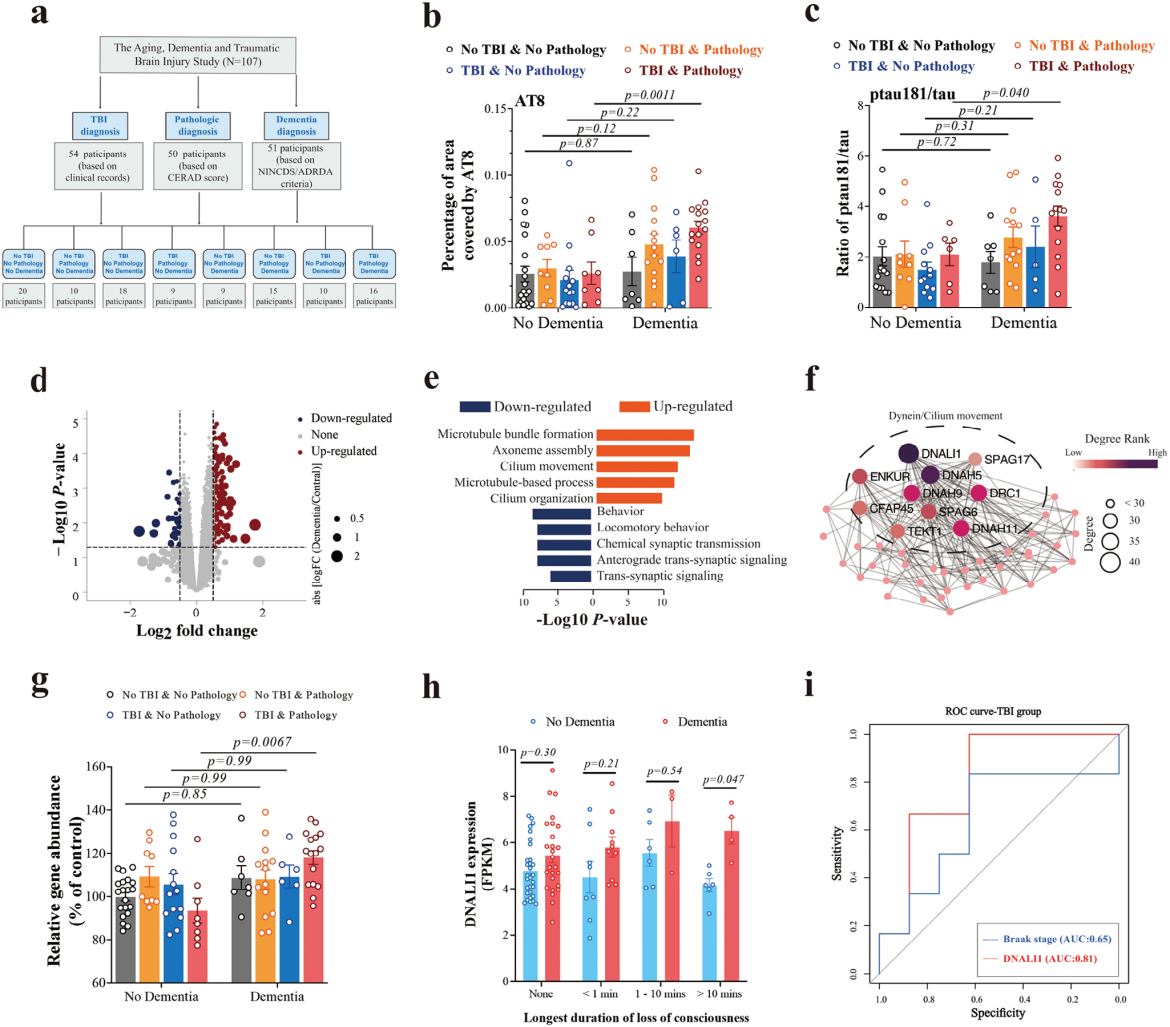
*DNALI1* is the critical gene of post‐TBI neurodegeneration. a) Schematic illustration of the participants' grouping. Participants were divided according to TBI diagnosis, dementia pathological diagnosis, and dementia diagnosis. b,c) Comparison of the percentage of the area covered by AT8 (b) using histology and immunohistochemistry (IHC) and the ratio of ptau181/tau detected by Luminex assays across different groups (c). d) Sample volcano plot for participants showing –log10 (*p*‐value) and log_2_FC values for all genes, highlighting those significantly upregulated (red dots) or downregulated (blue dots) genes with dementia; non‐significant genes are marked in gray. e) Reactome enrichment for upregulated (Log (Dementia/Non‐dementia group) >0.5, and *p <* 0.05) and downregulated (Log (Dementia/Non‐dementia group) <0.5, and *p <* 0.05) genes, based on Metascape. f) Protein‐protein interaction (PPI) analysis for the MEcyan module, where node size and color represent the degree rank and degree. g) Levels of *DNALI1* detected by RNA‐seq between dementia and non‐dementia groups. h) Comparison of *DNALI1* expression fragments per kilobase per million (FPKM) between dementia and non‐dementia groups across different durations of loss of consciousness. i) Receiver‐operating curve (ROC) analysis to determine the discriminative power of *DNALI1* expression to distinguish dementia and control in participants with TBI, with Braak stage score as a reference. The data are presented as means ± SEM. Two‐way ANOVA with Sidak's multiple comparisons test (b,c,g,h) was used. *p*‐values are indicated on the graphs.

We obtained clinical and genetic data from 107 individuals participating in the Aging, Dementia, and Traumatic Brain Injury Study.^[^ ^12^ ^]^ The study's demographics and characteristics are shown in Table S1 (Supporting Information). We categorized participants based on TBI diagnosis, dementia pathology, and dementia clinical diagnosis defined by the original study^[^ ^12^ ^]^ (**Figure** **1**a). Utilizing sequencing data from four brain regions (temporal cortex [TCx], parietal cortex [PCx], frontal white matter [FWM], and hippocampus [HIP]) we conducted a preliminary analysis employing principal component analysis, and revealing distinct gene expression patterns in the hippocampus compared to other brain regions (Figure S1a, Supporting Information). Subsequently, we performed an extensive analysis of AT8 levels (mapping tau phosphorylation at Ser202 or Thr205; Figure 1b; Figure S1b–d, Supporting Information) and the brain ptau181/tau ratio (Figure 1c; Figure S1e–g, Supporting Information) across the brain regions described above, comparing “clinical dementia” and “non‐demented” cohorts in TBI and pathology groups. Both AT8 and ptau181/tau ratio exhibited significant differences between “clinical dementia” and “non‐demented” groups exclusively within the hippocampus (Figure 1b,c), suggesting that this brain region may be the primary area affected by TBI. We have also examined changes related to Aβ pathology, detecting no significant differences among groups (Figure S2, Supporting Information), thus indicating that the pathology in the study may be consistent with CTE.

We next examined the transcriptome changes in the cohort, employing bioinformatics analysis specifically within participants originally described as “diagnosed with TBI” and with “dementia pathology”. We then compared “clinical dementia” and “non‐demented” groups (Figure 1d–f; Figure S3, Supporting Information). Through the enrichment of differentially expressed genes (Figure 1d; Figure S3a, Supporting Information), we identified microtubule‐, axoneme‐, and cilium‐related pathways as the most upregulated (Figure 1e), consistent with the results from Gene Set Enrichment Analysis (GSEA) (Figure S3b, Supporting Information). Furthermore, all genes were categorized into modules according to their expression levels and correlation coefficients. We calculated the *p*‐values for gene modules associated with dementia diagnosis and Braak. The module most relevant to these phenotypes (Figure S3c, Supporting Information, Diagnose: r = 0.58, *p =* 0.003; Braak: r = 0.54, *p =* 0.006) underwent further Gene Ontology (GO) analysis (String, version 11.5) (Figure S3d, Supporting Information). The results mirrored those observed in the pathways shown in Figure 1e, underscoring the importance of these pathways.

Through the analysis of degree rank results in the protein‐protein interaction (PPI) network, we further identified Axonemal dynein light intermediate polypeptide 1 (DNALI1) as the most critical gene in the gene module (Figure 1f, Degree = 42). Indeed, *DNALI1* expression was significantly elevated in the “clinical dementia” group (TBI diagnosis and dementia pathology) (Figure 1g, *p =* 0.0067). When comparing *DNALI1* expression at different durations of loss of consciousness, we observed a significant difference between the “clinical dementia” and “non‐demented” groups, particularly when the duration of loss of consciousness exceeded 10 min (Figure 1h, *p =* 0.047). Importantly, *DNALI1* expression was significantly correlated with AT8 (Figure S4a, Supporting Information, r = 0.53, *p =* 0.011) and the ratio of ptau181/tau (Figure S4b, Supporting Information, r = 0.68, *p =* 0.002) in participants with TBI‐“clinical dementia”, whereas such correlations were absent in “non‐demented” participants. Furthermore, the expression of *DNALI1* in TBI participants exhibited great predictive ability for dementia, which was even better than that of the Braak stage score (Figure 1i, AUC = 0.81). Collectively, these findings indicate that *DNALI1* is a critical gene for the development of neurodegeneration after TBI.

### 2.2 Altered DNALI1 Protein in In Vitro and In Vivo TBI Models

**Figure 2:**
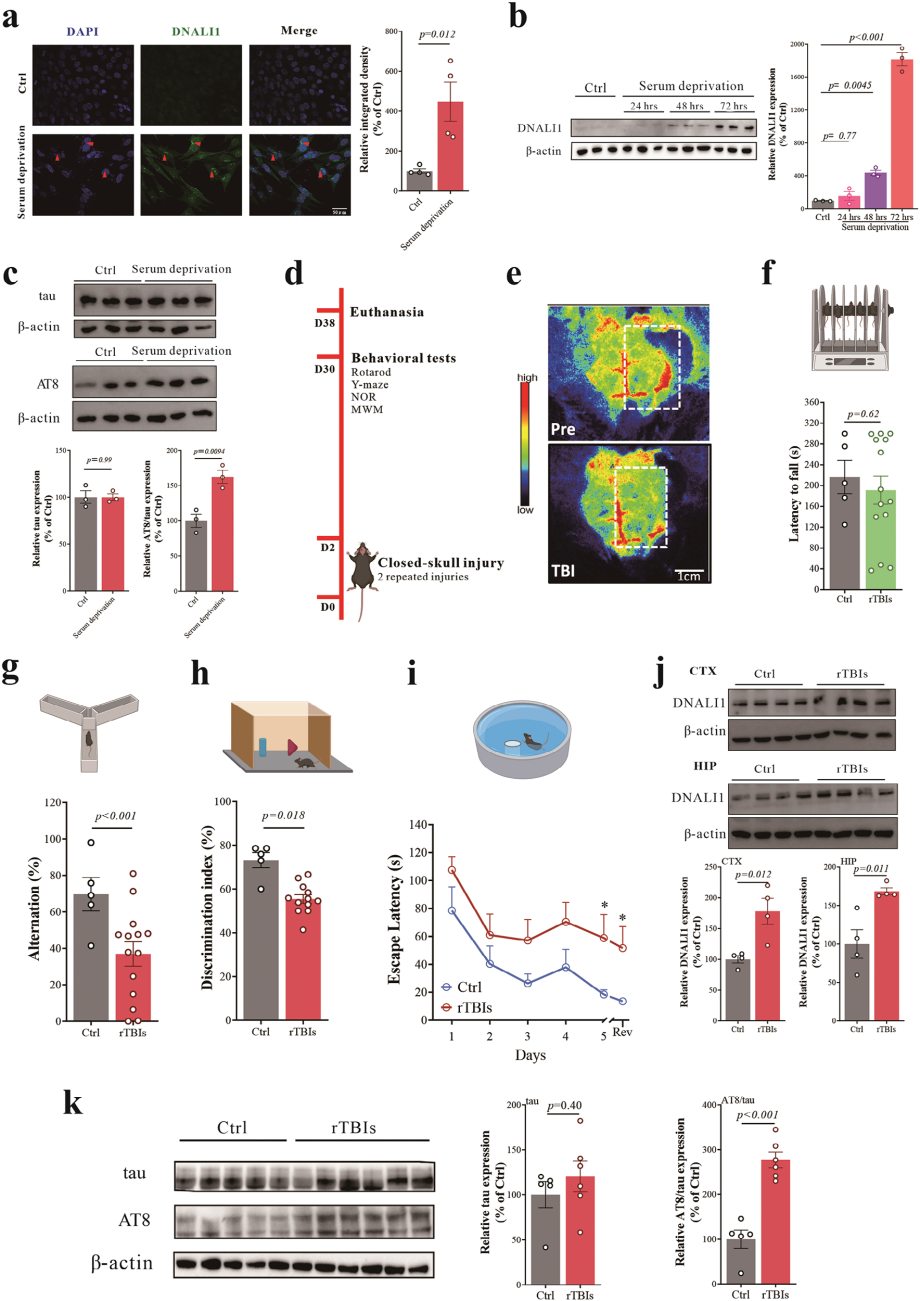
DNALI1 and tau pathology were changed significantly in vitro and in vivo. a) Immunofluorescent co‐labeling of DNALI1 (green) and nuclear (blue) with corresponding statistical results comparing control and serum deprivation models. *n* = 4 wells from one representative of four independent experiments. Scale bar, 50 µm, as indicated. b) Western blot analysis of DNALI1 in cells subjected to varying duration of serum deprivation (*n* = 3). Data are normalized to β‐actin and expressed relative to the control. c) Western blot analysis of tau and AT8 for serum deprivation model (*n* = 3). Data are normalized to β‐actin and expressed relative to the control. d) Diagrammatic drawing of the repeated mild closed‐head model and subsequent experiments. e) Blood flow changes in the TBI model before and after injury detected by Laser speckle imaging. Perfusion is visualized as a 2D color‐coded map of blood flow (red = high; blue = low), with a scale bar = 1 cm. f) Performance on the rotarod test analyzed one month after the brain injury. Ctrl, *n* = 5; TBI, *n* = 14. g) Performance on the Y‐Maze spontaneous alternation test analyzed one month after the brain injury. Ctrl, *n* = 5; TBI, *n* = 14. h) Performance on the Novel object recognition test analyzed one month after the brain injury. Ctrl, *n* = 5; TBI, *n* = 14. i) Performance on the Morris water maze test analyzed one month after the brain injury. Ctrl, *n* = 5; TBI, *n* = 14; Rev: Reversal learning. j) Western blot analysis of DNALI1 between control and TBI in the cortex and hippocampus (*n* = 4). Data are normalized to β‐actin and expressed relative to the control. k) Western blot analysis of tau and AT8 between control and TBI in the hippocampus. Control (Ctrl, *n* = 5; TBI, *n* = 6). Data are normalized to β‐actin and expressed relative to the control. Data are represented as the mean ± SEM. T‐test (a,c,f–k) and one‐way ANOVA with Tukey's multiple comparisons test (b) were used. *p*‐values are indicated on the graphs.

DNALI1 is a component of axonemal dynein, responsible for transporting cargo along the axoneme of eukaryotic cilia and flagella in conjunction with other components.^[^ ^13^ ^]^ Despite limited studies connecting DNALI1 to brain diseases, we explored whether DNALI1 expression is altered under conditions mimicking TBI. Serum deprivation has been previously used to simulate neuronal stress after TBI,^[^ ^14^ ^]^ and our immunofluorescence assay revealed a significant increase in DNALI1‐positive staining (**Figure** **2**a, *p* *=* 0.012), which was further confirmed by western blot analysis. Prolonged serum deprivation further amplified DNALI1 expression (Figure 2b), indicating its responsiveness to neuronal stress. Considering the previous correlation between AT8 and DNALI1 in human patients, we examined tau expression and phosphorylation. The expression of tau was unchanged (Figure 2c, *p =* 0.99), while the AT8/tau ratio increased (Figure 2c, *p =* 0.0094), aligning with previous findings (Figure S4a, Supporting Information).

Our analysis highlights the predominance of tau pathology in the human study, with Abeta pathologies remaining unaltered. To further investigate, we established an animal model of repetitive traumatic brain injuries (rTBIs) mimicking CTE. We employed closed‐skull, two mild replicate hit models with specific hit parameters (3.0 m s^−1^ strike velocity, 1.0 mm strike depth, and 500 ms) and an experiment time after injury of 30 days, (Figure 2d) based on a literature survey^[^ ^15^ ^]^ and pre‐experimentation (Figure S5, Supporting Information). We monitored blood flow using laser speckle imaging and assessed changes in cognitive performance in the model. Blood flow in the brains of mice significantly decreased after injury (Figure 2e), confirming the success of the model. Under the parameters used in this study, the rTBIs model did not induce motor dysfunction, as evidenced by the unaltered latency to fall in the rotarod test compared to controls (Figure 2f, *p =* 0.62). Conversely, mice exhibited significant cognitive impairment, as evidenced by significantly reduced alternation in the Y‐maze (Figure 2g, *p <* 0.001), reduced discrimination index in the novel object recognition (NOR) test (Figure 2h, *p* *=* 0.018), and prolonged escape latency in the Morris water maze (MWM) (Figure 2i). Subsequent to modeling, we found significant increases in DNALI1 expression in both the cortex and hippocampus (Figure 2j, cortex, *p =* 0.012; hippocampus, *p =* 0.011), along with an increased AT8/tau ratio (Figure 2k, *p <* 0.001).

### 2.3 *DNALI1* Knockdown Prevents Neurodegeneration after Repeated Mild Closed‐Head Injury

**Figure 3:**
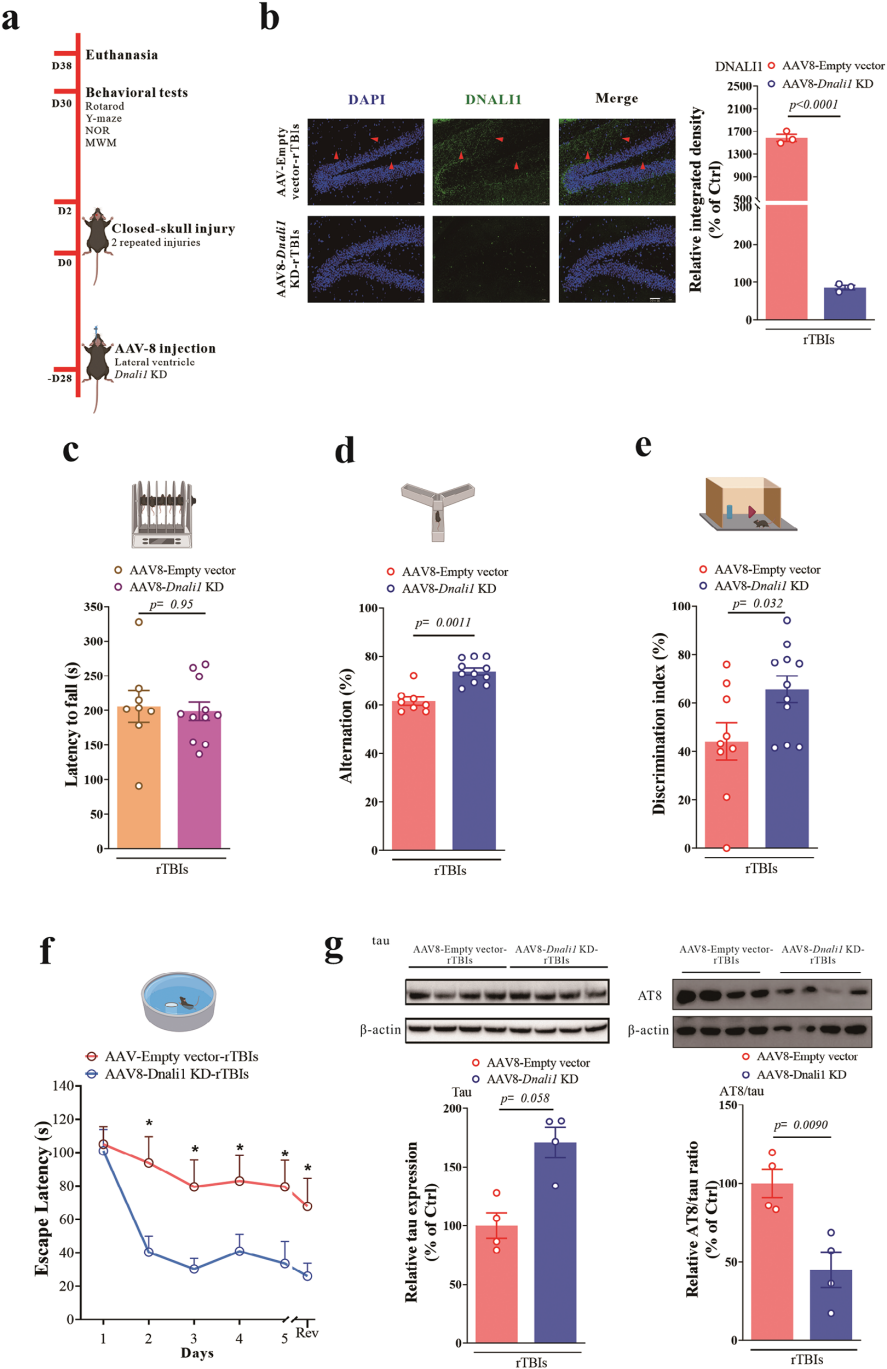
The knockdown of DNALI1 relieved cognitive impairment and the tau pathology of CTE. a) Diagrammatic representation of the brain AAV8‐DNALI1‐KD mice and subsequent experiments. b) Immunofluorescent co‐labeling of DNALI1 (green) and nuclei (blue) with corresponding statistical results after AAV injection and injury. *n* = 3. Scale bar, 50 µm, as indicated. c) Performance on the rotarod test analyzed after AAV injection and injury (AAV8‐empty‐TBI, *n* = 9; AAV8‐*Dnali1* KD‐TBI, *n* = 11). d) Performance on the Y‐Maze spontaneous alternation test analyzed after AAV injection and injury (AAV8‐empty‐TBI, *n* = 9; AAV8‐*Dnali1* KD‐TBI, *n* = 11). e) Performance on the Novel object recognition test analyzed after one after AAV injection and injury (AAV8‐empty‐TBI, *n* = 9; AAV8‐*Dnali1* KD‐TBI, *n* = 11). f) Performance on the Morris water maze test analyzed after AAV injection and injury (AAV8‐empty‐TBI, *n* = 9; AAV8‐*Dnali1* KD‐TBI, *n* = 11). g) Western blot analysis of tau and AT8 after AAV injection and injury in the hippocampus (*n* = 4). Data are normalized to β‐actin and expressed relative to the control. The data are represented as the mean ± SEM. T‐test (b–g) was used. *p*‐values are indicated on the graphs.

To determine whether DNALI1 contributes to neurodegeneration induced by repeated mild closed‐head injuries, we knocked‐down *DNALI1* in the brain by employing stereotaxic injection of adeno‐associated virus (AAV8)‐EF‐Cas9 + AAV8‐mDNALI1‐sp.g3 (1.0 × 10^12 GC mL^−1^) or AAV8 empty vectors as the control (**Figure** **3**a). This injection was administered 14 days before inducing head injury. Immunofluorescence analysis confirmed the efficacy of AAV8‐mDNALI1‐sp.g3 in attenuating the increase in DNALI1 protein expression after head injury (Figure 3b, *p <* 0.0001). Subsequent motor and cognitive performance assessments revealed that the knockdown of *DNALI1* did not alter motor function (Figure 3c, *p <* 0.95) but successfully rescued the cognitive impairment resulting from repeated mild closed head injury (Figure 3d–f). Consistent with this finding, western blot analysis of total tau and its phosphorylation revealed that the AT8/tau ratio significantly decreased after AAV injection (Figure 3g, *p =* 0.0090), consistent with the rescue of cognitive impairment. Taken together, these results suggest that targeting *DNALI1* after a head injury may offer a promising avenue to prevent further neurodegeneration.

### 2.4 DNALI1 Affects the Autophagosome‐Lysosome Fusion and Autophagic Flux in TBI

**Figure 4:**
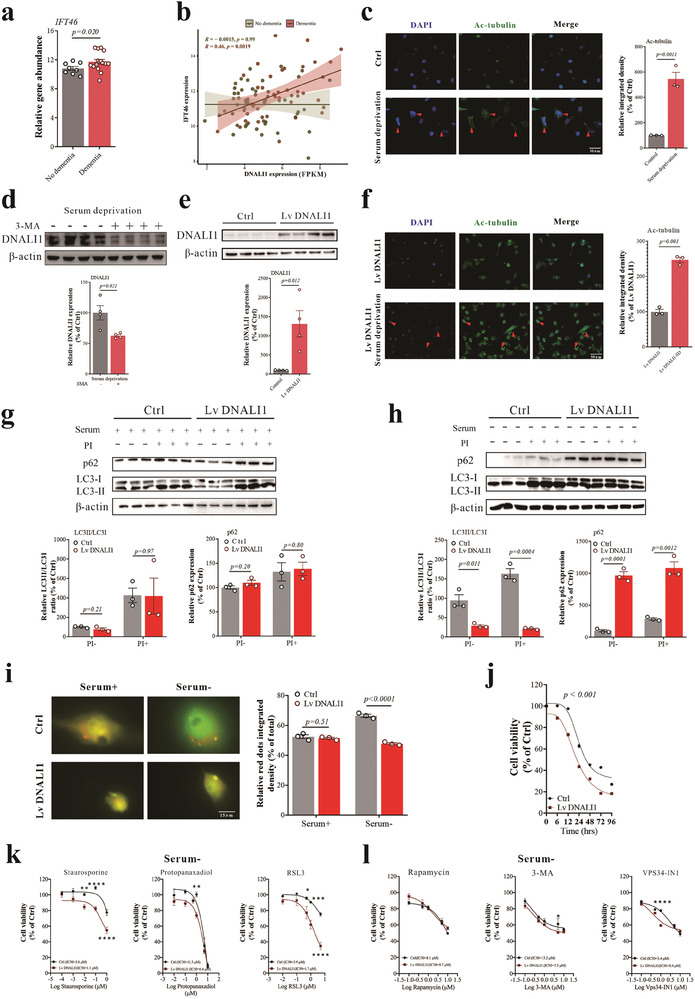
The increase of DNALI1 reduced the autophagic flux in pathological status. a) Levels of *IFT46* detected by RNA‐seq between dementia and non‐dementia groups. b) Correlation between *DNALI1* expression and *IFT46* expression for dementia and non‐dementia groups. c) Immunofluorescent co‐labeling of Ac‐tubulin (green) and nuclei (blue) with corresponding statistical results after 72 h of serum deprivation. *n* = 3. Scale bar, 50 µm, as indicated. d) Western blot analysis of DNALI1 after 3‐MA pretreatment under serum deprivation status (*n* = 4). Data are normalized to β‐actin and expressed relative to the control. e) Western blot analysis of DNALI1 after infection by lentivirus packaged with DNALI1 overexpression plasmid (*n* = 4). Data are normalized to β‐actin and expressed relative to the control. f) Immunofluorescent co‐labeling of Ac‐tubulin (green) and nuclei (blue) with corresponding statistical results after DNALI1 overexpression. *n* = 3. Scale bar, 50 µm, as indicated. g) Western blot analysis of LC3 and p62 for normal and DNALI1 overexpression cells after protease inhibitors (20 mm NH_4_Cl and 100 µm Leupeptin) pretreatment under normal serum conditions (*n* = 3). Data are normalized to β‐actin and expressed relative to the control. h) Western blot analysis of LC3 and p62 for normal and DNALI1 overexpression cells after protease inhibitors (20 mm NH_4_Cl and 100 µm Leupeptin) pretreatment under serum deprivation status (*n* = 3). Data are normalized to β‐actin and expressed relative to the control. i) Fluorescence levels for normal and DNALI1 overexpression cells after transfection of mCherry–GFP–LC3 plasmid under normal or serum deprivation status. *n* = 3. Scale bar, 15 µm, as indicated. j) Cell viability of normal and DNALI1 overexpression cells at 0, 6, 12, 24, 48, 72, and 96 h after serum deprivation, *n* = 6 wells from one representative of three independent experiments. k) Cell viability of normal and DNALI1 overexpression cells after treatment with apoptosis activator staurosporine, necrosis activator protopanaxadiol, and ferroptosis activator RSL3 treatment under 24 h of serum deprivation pretreatment status, *n* = 6 wells from one representative of three independent experiments. l) Cell viability of normal and DNALI1 overexpression cells after Rapamycin, 3‐MA, or VPS34‐IN1 treatment under 24 h of serum deprivation pretreatment status, *n* = 6 wells from one representative of three independent experiments. Data are represented as the mean ± SEM. T‐test (a,c–f), Pearson Correlation (b), or two‐way ANOVA with Sidak's multiple comparisons test (g–l) were used. *p*‐values are indicated on the graphs.

Given the primary involvement of DNALI1 in axonal transport, particularly in powering the beating of cilia through axonemal dynein,^[^ ^16^ ^]^ we examined differences in intraflagellar transport (IFT)‐related genes between “clinical dementia” and “non‐demented” in the Aging, Dementia and Traumatic Brain Injury Study. Notably, IFT protein 46 (*IFT46*), a component of the IFT complex, was significantly increased in participants with “clinical dementia” (**Figure** **4**a, *p =* 0.020), accompanied by a significant positive correlation with *DNALI1* (Figure 4b, r = 0.46, *p =* 0.0019). This correlation was not observed in non‐dementia participants, suggesting that *DNALI1* may regulate *IFT46* in the context of the disease.

IFT46, recognized as a core component of the intraflagellar transport machinery, plays a role in ciliogenesis. It is crucial for the IFT complex's movement along the microtubule‐based axoneme of cilia and flagella, transporting proteins and other molecules necessary for the assembly and maintenance of these structures during ciliogenesis.^[^ ^17^ ^]^ Indeed, the presence of detectable primary cilia, indicative of changes in ciliogenesis, increased by 400% 72 h after serum removal (Figure 4c, *p =* 0.0011).

Previous reports have indicated that ciliogenesis regulates autophagy via the Hedgehog pathway, inducing autophagy by directly acting on essential autophagy‐related proteins strategically located at the cilium's base through ciliary trafficking proteins.^[^ ^17a^ ^]^ Therefore, we hypothesized that DNALI1 participated in autophagy as part of the ciliogenesis process. We identified markers of autophagy after serum deprivation, including an elevated LC3II/LC3I ratio, decreased p62 expression (Figure S6a, Supporting Information), an increased number of autophagic vesicles (Figure S6b, Supporting Information, *p <* 0.001), and a notably elevated fluorescence intensity of LC3 (Figure S6c, Supporting Information). Meanwhile, we found that DNALI1 expression can be reduced by pretreatment with the autophagy inhibitor 3‐MA (1 µm, 6 h) during serum deprivation (Figure 4d, *p =* 0.021). This suggests that DNALI1 may be functional within the autophagy process.

To further clarify the role of DNALI1 in autophagy, we employed lentiviruses to overexpress *DNALI1* (Lv DNALI1) (Figure 4e, *p =* 0.012). This aggressive overexpression of *DNALI1* led to a significant increase in ciliogenesis (Figure 4f, *p =* 0.001), potentially impacting autophagic flux. The characteristics of autophagic flux (LC3II/LC3I ratio and p62 expression) exhibited no significant changes between the control and DNALI1 overexpression cells when serum was added, with or without 6 h pretreatment of protease inhibitors (20 mm NH_4_Cl and 100 µm Leupeptin) (Figure 4g). Protease inhibitors block the degradation of autophagic cargo, promoting autophagic flux (changes in LC3‐II content after blocking lysosomal degradation).^[^ ^18^ ^]^ However, we observed a significant reduction in autophagic flux upon serum removal in the DNALI1 overexpression cells (Figure 4h), indicating that the impact of elevated DNALI1 was specific to pathological conditions, such as nutrient deprivation.

To further verify this, we transfected cells with a pH‐sensitive reporter (mCherry–GFP–LC3; a fusion of mCherry, green fluorescent protein, and LC3) that highlighted autophagosomes as yellow puncta and autophagolysosomes (post‐lysosomal fusion) as red puncta. We found that the basal levels of autophagic vacuoles were comparable in control and DNALI1 overexpression cells, consistent with previous western blotting results. However, upon serum removal, cells overexpressing DNALI1 exhibited a significant reduction in autophagolysosome content (Figure 4i, *p <* 0.001). The impact of DNALI1 on autophagosome‐lysosome fusion and autophagic flux also affected cell viability following serum deprivation, with DNALI1 overexpression rendering cells more susceptible (Figure 4j, *p <* 0.001).

Autophagy is also associated with several cell death pathways, including apoptosis,^[^ ^19^ ^]^ necroptosis,^[^ ^20^ ^]^ and ferroptosis.^[^ ^21^ ^]^ Inhibition of autophagy can lead to the accumulation of damaged organelles, prompting autophagic vacuolization and ultimately triggering cell death pathways.^[^ ^22^ ^]^ Here, we found that DNALI1 overexpression also affected cell susceptibility to these cell death pathways under conditions of serum deprivation (Figure 4k), while such effects were not observed in normal serum conditions (Figure S7a, Supporting Information). Additionally, we analyzed the influence of DNALI1 overexpression on autophagy regulators. However, we only observed significant differences when VPS34‐IN1 was used (Figure 4l), likely due to its potent inhibitory effect on both VPS34 and DNALI1. Importantly, the normal serum status did not alter the serum levels (Figure S7b, Supporting Information).

**Figure 5:**
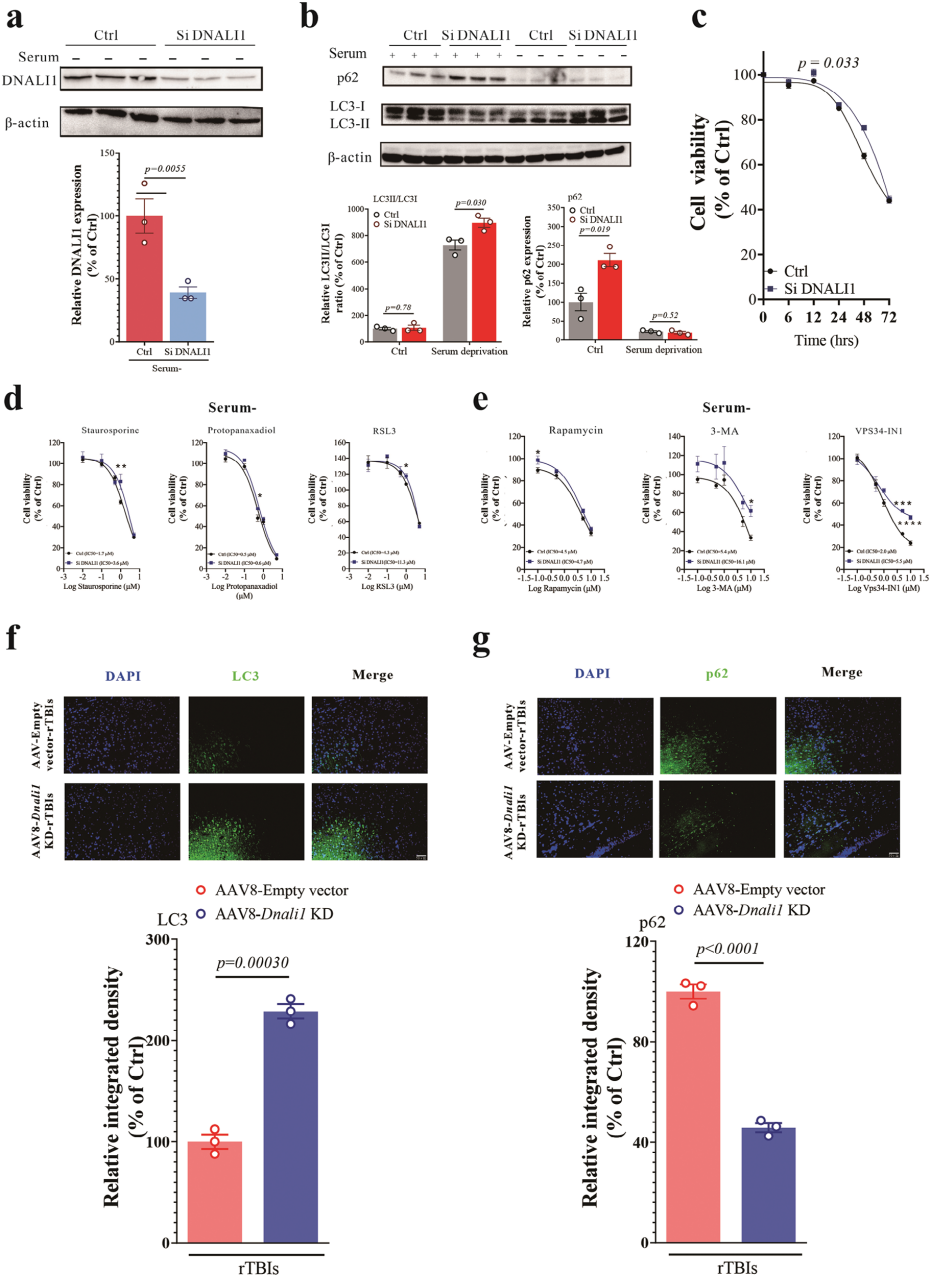
The decrease of DNALI1 increased the autophagic flux in pathological conditions. a) Western blot analysis of DNALI1 after siRNA transfection (*n* = 3). Data are normalized to β‐actin and expressed relative to the control. b) Western blot analysis of LC3 and p62 for normal and si‐DNALI1 cells under normal or serum deprivation status, *n* = 3. Data are normalized to β‐actin and expressed relative to the control. c) Cell viability of normal and si‐DNALI1 cells at 0, 6, 12, 24, 48, and 72 h after serum deprivation, *n* = 6 wells from one representative of three independent experiments. d) Cell viability of normal and si‐DNALI1 cells after treatment with apoptosis activator staurosporine, necrosis activator protopanaxadiol, and ferroptosis activator RSL3 under 24 h of serum deprivation pretreatment status, *n* = 6 wells from one representative of three independent experiments. e) Cell viability of normal and si‐DNALI1 cells after Rapamycin, 3‐MA, or VPS34‐IN1 treatment under 24 h of serum deprivation pretreatment status, *n* = 6 wells from one representative of three independent experiments. f) Immunofluorescent co‐labeling of LC3 (green) and nuclei (blue) with corresponding statistical results after AAV injection and injury. *n* = 3. Scale bar, 50 µm, as indicated. g) Immunofluorescent co‐labeling of p62 (green) and nuclei (blue) with corresponding statistical results after AAV injection and injury. *n* = 3. Scale bar, 50 µm, as indicated. Data are represented as the mean ± SEM. T‐test (a,f,g), or two‐way ANOVA with Sidak's multiple comparisons test (b–e) were used. *p*‐values are indicated on the graphs.

To further validate the effect of DNALI1 on autophagy, we employed siRNA to inhibit DNALI1 expression (Si DNALI1) (**Figure** **5**a, *p =* 0.0055). Autophagic flux, as indicated by the LC3II/LC3I ratio and p62 expression, significantly increased during serum deprivation, whereas no such effect was observed under normal serum conditions (Figure 5b). The effects of DNALI1 on autophagic flux also affected cell viability following serum deprivation, with cells exhibiting reduced DNALI1 displaying increased resistance (Figure 5c, *p =* 0.033). Moreover, we also found that DNALI1 reduction affected cell resistance to cell death and autophagy regulators under serum‐deprived conditions (Figure 5d,e). In contrast, only autophagy regulators were affected in normal serum (Figure S8, Supporting Information). Additionally, experiments conducted using a previously constructed AAV8‐*Dnali1* KD animal model showed that LC3 and p62 immunofluorescence results indicated a significant increase in autophagic flux upon *Dnali1* KD (Figure 5f,g).

## 3 Discussion

**Figure 6:**
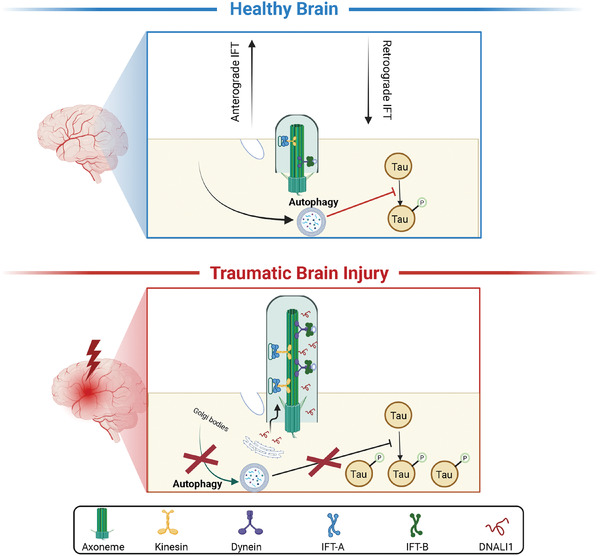
Working hypothesis of the role of DNALI1 in post‐TBI neurodegeneration. Following TBI, ciliogenesis increases under mechanical and oxidative stress, leading to the increase of DNALI1. DNALI1 prevents the clearance of phosphorylated tau by autophagy, inhibiting autophagic flux and contributing to the development of CTE. This figure was generated using BioRender.com.

CTE, occurred after repetitive TBIs with hyperphosphorylated tau accumulation in the hippocampal region, is yet to be cured. In this study, through comprehensive screening of data derived from human samples and subsequent validation in cells and rodent models of TBI, we demonstrated that DNALI1 is a critical factor in CTE, operating through a decrease in autophagic flux. During CTE, ciliogenesis increases in response to external mechanical and oxidative stress, along with elevated DNALI1 levels, resulting in a decrease in autophagy levels. This convergence of diverse physiopathological processes creates a blockade in autophagic function, impeding the clearance of damaged organelles and deleterious material, thereby increasing phosphorylated tau protein levels and ultimately precipitating cognitive impairment. Notably, both autophagic dysfunction and cognitive decline after TBI were alleviated by *DNALI1* knockdown (**Figure** **6**).

Previous studies have highlighted a noteworthy discordance between clinical and final neuropathological diagnoses in more than one‐third of the cases.^[^ ^23^ ^]^ Moreover, older individuals who remained cognitively intact proximate to death often exhibited significant AD neuropathologic changes.^[^ ^24^ ^]^ Additionally, a significant proportion (∼15–30%) of patients with mild cognitive impairment (MCI) manifest evidence of neurodegeneration without amyloid deposition.^[^ ^25^ ^]^ Given these complexities, we categorized participants based on their TBI diagnosis, dementia pathology, and clinical dementia diagnosis. However, while acknowledging the significance of TBI severity in this study, we were constrained by the data provided and the number of participants, preventing further subgrouping based on the severity. In addition, the differential diagnosis between CTE and AD or other types of dementia is also crucial.^[^ ^26^ ^]^ In Aging, Dementia, and Traumatic Brain Injury Study, we categorized participants based on the original described TBI diagnosis, dementia pathology, and dementia clinical diagnosis. However, based on the reported history of TBI and the sole presentation of tau pathology, the cohort is more likely with CTE. In the subsequent animal experiments, a model mimicking CTE was also applied, revealing similar pathological features. Therefore, there is a clear need for improved accuracy of clinical diagnostic criterias to differentially diagnosis CTE and AD.

We identified the hippocampus as a key brain region in post‐traumatic cognitive impairment, contrasting its significance to the PCx, TCx, and FWM based on distinct gene expression patterns and differential levels of tau phosphorylation. Nevertheless, it is essential to acknowledge that other brain regions also play significant roles in cognition. For instance, the prefrontal cortex is integral to executive functions, such as decision‐making, planning, and working memory. Meanwhile, the TCx and PCx contribute to various aspects of sensory perception, language processing, and attention.^[^ ^27^ ^]^ Therefore, our future research will continue to explore the intricate relationships between diverse brain regions and cognitive impairment following TBI.

In this study, we successfully identified ciliogenesis and ciliary signaling pathways as potential contributors to cognitive impairment following TBI. Previous studies have indicated a significant increase in the length of primary cilia in the hippocampus of APP/PS1 compared to controls, and intraperitoneal administration of 5‐hydroxytryptamine receptor subtype 6 (5‐HT_6_) antagonist SB271046 effectively reduced cilia length and rescued cognitive impairment in APP/PS1 mice.^[^ ^28^ ^]^ Among these, DNALI1, an essential component of the ciliated dynamic arm,^[^ ^29^ ^]^ emerged as the key gene in our analysis, primarily responsible for cilium movement. Our findings shed light on the mechanistic underpinnings of the relationship among TBI, DNALI1, and ciliogenesis, evident through the increased presence of cells with detectable primary cilia in both the in vitro TBI model (Figure 4c) and the DNALI1 overexpression model (Figure 4f). In a human brain sequencing study,^[^ ^30^ ^]^ *DNALI1* exhibited a threefold higher expression in frontotemporal lobar degeneration with ubiquitinated inclusions (FTLD‐U) compared to controls. Furthermore, DNALI1 interacted with the major component of ubiquitinated inclusions in FTLD‐U and TDP‐43.^[^ ^31^ ^]^ Given TDP‐43′s implication in synaptic and cognitive deterioration following TBI,^[^ ^32^ ^]^ our work may offer insights into TDP‐43 changes, where autophagy regulates TDP‐43 through interactions with autophagosomes mediated by autophagy‐associated proteins, such as LC3 and p62, leading to its degradation.^[^ ^33^ ^]^ Another transcriptome sequencing study, focusing on middle temporal gyrus tissue from 100 patients with AD and controls, also revealed that *DNALI1* expression in AD cases was significantly higher than in controls. Moreover, a significant positive correlation was observed between *DNALI1* and NFTs density,^[^ ^34^ ^]^ a correlation consistent with our findings here. Collectively, our results position DNALI1 as a critical protein implicated in cognitive impairment.

In our pursuit of understanding neurodegeneration after TBI, we also implemented a robust animal model (Figure 2e–i) that accurately recapitulates TBI‐induced cognitive deficits. While establishing a TBI model is relatively straightforward, the careful selection of study parameters has been identified as a key factor contributing to the observed heterogeneity in studies conducted across different laboratories.^[^ ^35^ ^]^ Factors such as the number, severity, and timing of repeated concussive events also demand meticulous consideration. After testing various models of TBI, we opted for a closed skull model to circumvent primary injury occurrence. We also determined the optimal number of repeated hits in the TBI model and pinpointed the post‐injury timeframe during which animals developed the most pronounced cognitive impairment, as discerned through changes in behavioral tests. These findings carry significant implications for the design and execution of future studies exploring TBI or CTE in animal models.

Autophagy, a self‐degradative process necessary for maintaining energy balance during critical developmental stages and in response to nutrient stress,^[^ ^36^ ^]^ has gained prominence for its role in preserving neuronal homeostasis.^[^ ^37^ ^]^ Most neurodegenerative diseases, characterized by abnormal protein aggregation, such as hyperphosphorylation of tau protein,^[^ ^38^ ^]^ are linked to autophagy dysfunction. Here, we demonstrate that DNALI1 affects cognition by regulating autophagy under pathological conditions. Notably, DNALI1 levels showed no influence on autophagy under normal conditions, as indicated by the absence of significant differences following DNALI1 overexpression or reduction. However, under serum deprivation conditions, DNALI1 overexpression reduced autophagy levels, whereas DNALI1 inhibition enhanced them. The modulatory role of DNALI1 in autophagy further contributes to cellular sensitivity to cell death pathways. Consistent with previous findings suggesting that increased autophagy protects cells from apoptosis,^[^ ^19^ ^]^ our results align with this protective mechanism. The ability of DNALI1 to regulate cell death pathways may have further implications for neurodegenerative diseases and other conditions where cell death is implicated. A recent study by Zhao et al. highlighted the impact of rTBIs on neuronal axonal microtubule assembly, leading to microtubule depolymerization and subsequent tau dissociation and spread throughout the brain.^[^ ^39^ ^]^ Our study suggests that DNALI1 may represent an upstream event, with its elevation triggering tau hyperphosphorylation, potentially contributing to the observed tau spread reported by Zhao et al.

In summary, our study strongly suggests that DNALI1 impacts the outcome of neurodegeneration after TBI by regulating autophagy. Targeting the excessive production of DNALI1 emerges as a promising approach to thwart the progression of CTE neuropathology, offering new prospects for therapeutic interventions in this challenging clinical context.

## 4 Experimental Section

### Reagents

Reagents were purchased from Sigma‐Aldrich unless otherwise specified.

### Data Acquisition and Study Design

Publicly available datasets were obtained from the Aging, Dementia, and Traumatic Brain Injury Study (http://aging.brain‐map.org/),^[^ ^12^ ^]^ which provides a systematic and extensive dataset encompassing specimen metadata, histology, immunohistochemistry, in situ hybridization, RNA sequencing, protein quantification, and isoprostane quantification for 107 individuals. Participants were categorized into eight groups based on TBI diagnosis, dementia pathology (CERAD>1), and clinical dementia diagnosis (using the NINCDS/ADRDA criteria).

### Bioinformatics Analysis

Differential expression was assessed using a linear model and the Bioconductor limma package^[^ ^40^ ^]^ in R, version 3.6.3 (R Foundation) based on a |fold change| ≥ 0.5 and *p*‐value < 0.05. The results were input into the Metascape database^[^ ^41^ ^]^ (https://metascape.org/gp/index.html) to explore Gene Ontology (GO) pathways. Heatmaps were generated using the Pheatmap package^[^ ^42^ ^]^ in R, version 3.6.3 (R Foundation), with z‐scores calculated for each gene row using the mean expression of biological replicates. Volcano plots, illustrating differentially expressed genes, were generated using the Enhanced Volcano package^[^ ^43^ ^]^ in R, version 3.6.3 (R Foundation). The network diagram for PPI was calculated and visualized using Cytoscape (https://www.cytoscape.org/), and hub genes were ranked using cytoHubba.^[^ ^44^ ^]^ Gene Set Enrichment Analysis (GSEA) (version 4.0.3, https://www.broadinstitute.org/gsea/)^[^ ^45^ ^]^ was employed to identify overrepresented GO pathways (MSigDB, version 7.1) for upregulated or downregulated genes. Co‐expression networks for RNA‐seq data and their correlation values with phenotypic data were constructed and calculated using the weighted gene co‐expression network analysis (WGCNA) package^[^ ^46^ ^]^ in R, version 3.6.3 (R Foundation).

### Cell Culture

The neuronal cell line STHdhQ7/Q7 (a gift from Dr. Boxun Lu, Fudan University) was cultured in Dulbecco's Modified Eagle's Medium (DMEM) supplemented with 10% fetal calf serum and 100 Units/ml penicillin/streptomycin (all purchased from Thermo Fisher Scientific, USA).

### In Vitro TBI Model

The serum deprivation model was employed to replicate neuronal stress after TBI, as previously described.^[^ ^14^ ^]^ Briefly, cells were seeded in 6/96‐well plates (5×10^4^ cells/mL). After plating, the cell culture medium was removed, washed three times with DPBS (Thermo Fisher Scientific, USA), and then replaced with a serum‐free medium. The duration of serum deprivation was adjusted according to experimental requirements, as indicated in the figure legends.

### Cell Viability Assay

Cells were seeded into 96‐well plates (5×10^4^ cells/mL) and treated with the selected compounds [Rapamycin (Cat No. S1039, Selleck Chemicals), 3‐Methyladenine (Cat No. S2767, Selleck Chemicals), VPS34‐IN1 (Cat No. S7980, Selleck Chemicals), RSL3 (Cat No. S8155, Selleck Chemicals), Staurosporine (Cat No. S1421, Selleck Chemicals), (20S)‐Protopanaxadiol (Cat No. S4746, Selleck Chemicals)] after plating. Cell viability was assessed at different time points after treatment (24 h unless otherwise specified) using the Cell Counting Kit‐8 (CCK‐8) cytotoxicity assay (Bimake, B34304), as previously described.^[^ ^47^ ^]^

### Transmission Electron Microscopy

After treatment, cells were collected into a 1.5 mL EP tube, and a low‐speed centrifugation step was employed to settle the cells at the bottom of the EP tube. Subsequently, the supernatants were removed. The collected cell pellets were fixed with 2.5% glutaraldehyde for 4 h at 4 °C and post‐fixed in 1% osmium tetroxide for 2 h at 20 °C. Then, they were dehydrated using a gradient of ethanol (50–100%) and acetone, embedded in epoxy resin, and polymerized for 48 h at 60 °C. Ultrathin sections (80 nm) were cut and stained with uranyl acetate and lead citrate prior to transmission electron microscopy (HT7700, HITACHI). Images were captured using a SlowScan CCD camera and the iTEM software (Ver 01.07, Olympus Soft Imaging Solutions). The quantification of autophagic bodies followed established procedures as previously described.^[^ ^48^ ^]^

### siRNA Transfection

Cells were seeded onto 6/96‐well plates (5×10^4^ cells/mL) and transfected with siRNA. The target sequences for the siRNA used in this study are shown in Table S2 (Supporting Information). The final siRNA concentration used was 10 nm, and the transfection was carried out using RNAiMax (Invitrogen, Cat No. 13778150) following the manufacturer's specifications. Follow‐up experiments were continued 24 h after transfection, as described previously.^[^ ^49^ ^]^

### Animals

Eight‐week‐old male C57BL/6 mice were obtained from Beijing HFK Bioscience Co., Ltd. and housed in a specific pathogen‐free facility at the State Key Laboratory of Biotherapy (Sichuan University, China). The mice underwent an adaptive feeding period of one week prior to the commencement of the experiments. All mouse‐related procedures were conducted in accordance with the Institutional Guidelines of the Animal Care and Use Committee (K2018071, Sichuan University, China).

### Repetitive Traumatic Brain Injuries

A mouse model involving closed skulls and repetitive mild head injuries was used, as described previously.^[^ ^15^, ^50^ ^]^ Briefly, mice were placed in a stereotaxic frame following anesthesia with isoflurane, 4% for induction and 1–2% for maintenance. The injury was induced using a 3 mm blunt metal impactor tip positioned at 1.8 mm caudal to bregma and 2.0 mm left of midline. The injury was triggered by an electromagnetically controlled cortical impact device (Custom Design & Fabrication, Inc, USA) with 3.0 m s^−1^ for strike velocity, 1.0 mm for strike depth, and 500 ms for dwell time to the exposed skull. Post‐impact, the skin was sutured, disinfected with iodophor, and the mice were allowed to recover from anesthesia on a warming pad before being returned to their home cages. A second identical injury procedure was performed 24 h later. Sham injuries followed the same procedures and anesthesia, excluding the delivery of an impact.

### Y‐Maze Spontaneous Alternation Test

The Y‐maze spontaneous alternation test was used to assess spatial working memory, as described previously.^[^ ^51^ ^]^ Briefly, one month after model establishment, each mouse was placed naive to the Y maze (39.5×8.5×13 cm, Sansbio co. ltd, China), at the same end of one arm and allowed to move freely through the maze during an 8‐minute session. The entire session was video‐recorded (Basler acA640‐120gm, Basler Vision Technology), and the number of arm entries for each mouse was subsequently calculated.

### Morris Water Maze Test

The Morris water maze (MWM) test was conducted to assess spatial learning, as previously described.^[^ ^52^ ^]^ Briefly, a circular water tank (diameter 80 cm) was filled with milk powder‐stained water, housing a concealed round platform (diameter 7 cm) positioned 1 cm below the water surface at the center of a specific quadrant. The test consisted of a place navigation test (five days) and a spatial probe test (one day). During the place navigation test stage, mice started from one of the four quadrants facing the pool wall and ended upon reaching the platform. If mice failed to locate the platform within 120 s, they were guided to it. In the subsequent spatial probe test, the platform was removed, and task performance was recorded for 120 s. Mouse movements in the water pool were recorded by a video camera (Basler acA640‐120gm, Basler Vision Technology), and task‐related metrics, including swimming paths, speed, and time spent in each quadrant, were recorded using WMT‐100 software (Chengdu Techman Software Co. Ltd, China).

### Novel Object Recognition Test

The novel object recognition (NOR) test was performed to evaluate memory retention, as described previously.^[^ ^52^ ^]^ Briefly, 24  h before the test, mice underwent a 5‐minute habituation period in the arenas (50 cm × 50 cm plastic container, Sansbio co. ltd, China) without objects. On the subsequent day, the mice re‐entered the arena from the same starting point (facing the bottom‐left corner) for the training and testing stages. In the first stage of the test, animals were confronted with two identical objects for 10 min. In the second stage, 1 h after the familiarization period, the animals were exposed to two dissimilar objects in the same open field for 5 min: one familiar object used in the first phase and another novel object. The time spent exploring each object in stage two was detected using Supermaze (ver 3.3, Shanghai XinRuan Information Technology co. ltd, China). The discrimination index (DI) was calculated using the following equation: DI = TN/ (TN + TF), where TN represents the exploration time devoted to the novel object and TF is the exploration time for the familiar object.

### Rotarod Treadmill Test

The rotarod treadmill test was conducted to assess motor coordination, as described previously.^[^ ^53^ ^]^ One month after establishing the model, animals were placed on a rotarod treadmill (RWD, Shenzhen, China) in the accelerating rotor mode (10 speeds ranging from 4 to 40 rpm for 5 min). The interval from the moment the animal mounted the rod to when it fell off was recorded as the retention time, and mice that remained on the accelerating rotating rod for 300 s were recorded as survivors. Animals underwent training for two days, with three trials per day, before model establishment, and the mean duration on the rod was recorded to obtain stable baseline values. Performance on the rotarod test was measured three times a month after repeated mild closed‐head injuries.

### Immunofluorescence

For immunofluorescence of brain tissue sections, mice were anesthetized with pentobarbitone and perfused with ice‐cold PBS (0.01 m, pH 7.4). Brains were isolated, fixed in 4% paraformaldehyde in PBS overnight, and then sectioned into 4 µm paraffin sections. After staining with primary antibodies (Anti‐DNALI1, Abcam, ab155490, 1:500; Anti‐Acetylated Tubulin, Proteintech, 66200‐1‐Ig, 1:250) and fluorescent‐tagged secondary antibodies (Alexa Fluor 488 Goat Anti‐Mouse IgG, Jackson ImmunoResearch, 115‐005, 1:500; Cy3 Goat Anti‐Mouse IgG, Jackson ImmunoResearch, 115–165, 1:500), the nuclei were counterstained with 4,6‐diamidino‐2‐phenylindole (DAPI), and coverslips were applied. The labeled sections were captured using a confocal laser‐scanning microscope (Nikon ECLIPSE Ti‐S). Brightness and contrast adjustments were uniformly applied to the captured images using CaseViewer software, version 2.4 (3DHIESTECH Ltd).

For immunofluorescence, cells were seeded on cell‐climbing slides. After treatment, cells were fixed with 4% paraformaldehyde at room temperature for 30 min. Subsequently, cells were blocked for 1 h at room temperature with 10% normal goat serum (Solarbio, SL038) dissolved in 0.2% Triton X‐100 PBS and incubated with the primary antibody (DNALI1, Abcam, ab155490, 1:500) overnight at 4 °C in a humidified chamber. The following day, the secondary antibody (Alexa Fluor 488 AffiniPure Alpaca Anti‐Rabbit IgG (H+L), Jackson ImmunoResearch, 611‐545‐215, 1:500) and DAPI were used for imaging. Images were captured under a fluorescence microscope (OLYMPUS VS200, JPN) and quantified using ImageJ (, version 1.5.1 (NIH, USA).

### Western Blot

Samples were homogenized in cell lysis buffer (P0013, Beyotime) supplemented with the protease inhibitor phenylmethylsulfonyl fluoride (1:100, ST507, Beyotime) and centrifuged at 12000 × g for 30 min. The supernatant was collected, and the total protein concentration was determined using a BCA protein assay kit (P0011, Beyotime). Equal amounts of protein were separated on 4–12% bis‐Tris gels with MOPs running buffer at 140 V for 1.5 h and then transferred to nitrocellulose membranes using a Trans‐Blot system at 100 V for 1 h. Then, the membranes were washed with 1 × TBST for 5 min at room temperature (20 °C), shaken, and blocked with 5% skim milk in 1 × TBST for 1 h at room temperature. The following antibodies were diluted in 1 × TBST as indicated: 1:3000 for anti‐LC3 (14600‐1‐AP, Proteintech), 1:3000 for anti‐P62 (18420‐1‐AP, Proteintech), 1:10 000 for anti‐mouse IgG (A9044, Sigma‐Aldrich), 1:10 000 for anti‐rabbit IgG (A0545, Sigma‐Aldrich), 1:2000 for anti‐DNALI1 (ab155490, Abcam & 17601‐1‐AP, Proteintech); 1:1000 for anti‐Tau5 (ab80579, Abcam); 1:1000 for Anti‐Phospho‐Tau (Ser202, Thr205) (MN1020, Thermo Fisher Scientific). All uncropped images of western blots are shown in Figure S9 (Supporting Information).

### Adeno‐Associated Viral Vector Construction and Preparation

To knockdown murine DNALI1 with a recombinant adeno‐associated virus (rAAV) in vivo, sgRNAs were designed using online tools (https://crispr.mit.edu/ and https://crispr.cos.uni‐heidelberg.de/). Off‐target effects were assessed using resources available at https://asia.ensembl.org/. sgRNA sequences with fewer off‐target sites were selected for further analysis. The target sequences of the sgRNA used in this study are shown in Table S3 (Supporting Information). The sgRNAs for murine DNALI1 knockdown were inserted into the plasmid, which was used to produce mDNALI1‐sp. g3‐rAAV8 (Obio Technology, Shanghai, China) was used to knockdown DNALI1, and the AAV8‐empty vector was used as a control. All rAAV8 vectors were generated using the triple‐plasmid co‐transfection method in human embryonic kidney 293 cells. After 72 h post‐transfection, the rAAV8 vectors were collected, purified through two rounds of CsCl gradient ultracentrifugation, followed by silver staining and genome copy titration. The viral vectors were aliquoted and stored at −80 °C before use.

### AAV Injection

For stereotaxic injection, mice were anesthetized by means of intraperitoneal injection of pentobarbital (100 mg k^−1^g, P11011, Bioreagent) and fixed on a stereotaxic plate (RWD, Shenzhen, China). A hole was drilled into the bone using a hand drill (RWD, Shenzhen, China). Eight microliters of the virus, with a total titer of 1.0 × 10^12^ genome copies, were injected into the lateral ventricle (AP = −0.6 mm, ML = −1.0 mm, DV = 2 mm) relative to the bregma. The needle was then held in place for an additional 10 min. After injection, the needle was withdrawn, and the wound was sutured.

### Statistical Analysis

Data are presented as individual values. Statistical analysis was conducted using GraphPad Prism 8.0 software (GraphPad Software, Inc., USA), and Student's *t*‐test was used to analyze statistical differences between two groups. One‐way ANOVA or repeated‐measures two‐way ANOVA with Tukey's post hoc test was used when appropriate. Values are presented as mean ± SEM, and individual data points represent individual samples or animals. Further details of the statistical analysis can be found in the figure legends. *p*‐value < 0.05 was considered statistically significant.

## Conflict of Interest

The authors declare no conflict of interest.

## Author Contributions

P.L. conceived and supervised the study. P.L. and X.D. designed and raised funds for this study. X.D. performed data analysis; X.D. and Q.W. performed cell biology experiments; X.D., B.D., S.C., and Y.C. performed animal experiments; W.L. coordinated the experiments; and X.D., K.L., S.Q., Q.Z.T., W.L., and P.L. integrated the data and drafted the manuscript. All authors have edited and approved the manuscript.

## Supporting information

Supporting Information

## Data Availability

The data that support the findings of this study are available from the corresponding author upon reasonable request.
