# Supplementary material for: DNALI1 Promotes Neurodegeneration after Traumatic Brain Injury via Inhibition of Autophagosome‐Lysosome Fusion
Source: Adv Sci (Weinh). 2024 Feb 13;11(15):2306399. doi: 10.1002/advs.202306399 (PMC11022701; doi:10.1002/advs.202306399)
Supplement: Supplementary file 1 — Supporting Information [file ADVS-11-2306399-s001.pdf]

ADVANCED SCIENCE
Open Access

Supporting Information

for *Adv. Sci.*, DOI 10.1002/advs.202306399

DNALI1 Promotes Neurodegeneration after Traumatic Brain Injury via Inhibition of Autophagosome-Lysosome Fusion

Xulong Ding, Shuqiang Cao, Qing Wang, Bin Du, Kefeng Lu, Shiqian Qi, Ying Cheng, Qing-zhang Tuo, Weibo Liang and Peng Lei**

Supporting Information

DNALI1 promotes neurodegeneration after traumatic brain injury via inhibition of autophagosome-lysosome fusion

Xulong Ding, Shuqiang Cao, Qing Wang, Bin Du, Kefeng Lu, Shiqian Qi, Ying Cheng, Qing-zhang Tuo, Weibo Liang[*], *Peng Lei*[*]

*Corresponding author. Email: peng.lei@scu.edu.cn (P.L.) or liangweibo@gmail.com (W-b.L.)

This PDF file includes:

Figures. S1 to S9
Tables S1 to S3

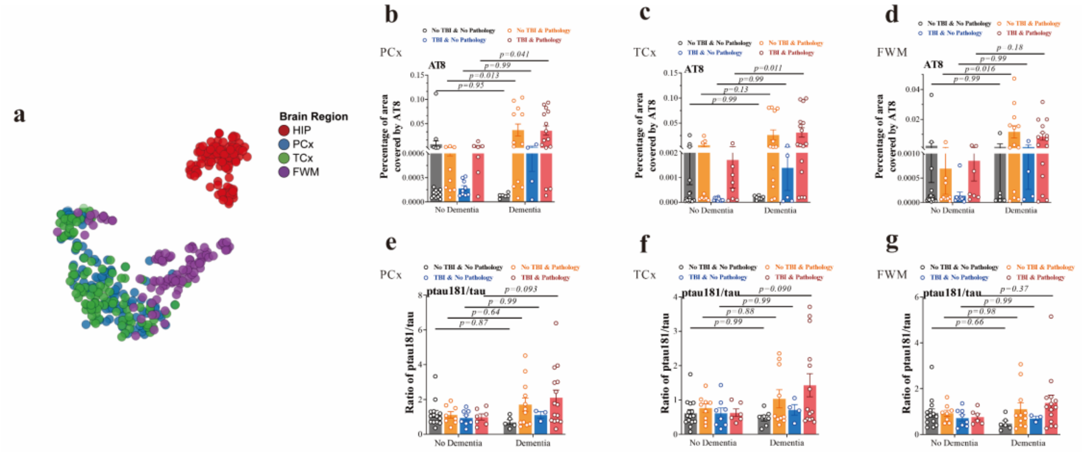

Figure S1. The hippocampus is the most unique brain region in the study. a. t-Distributed Stochastic Neighbor Embedding (tSNE) analysis for four brain regions of RNA-seq data in Aging, Dementia and Traumatic Brain Injury Study. **b-d.** The comparison of the percentage of area covered by AT8 detected by histology and immunohistochemistry (IHC) among different groups in the parietal cortex (PCx) (**b**), temporal cortex (TCx) (**c**) and frontal white matter (FWM) (**d**). e-g. The comparison of the ratio of ptau181/tau detected by Luminex assays among different groups in PCx (**e**), TCx (**f**) and FWM (**g**). Two-way ANOVA with Sidak's multiple comparisons test (**b-g**) was used. P values are indicated on the graphs.

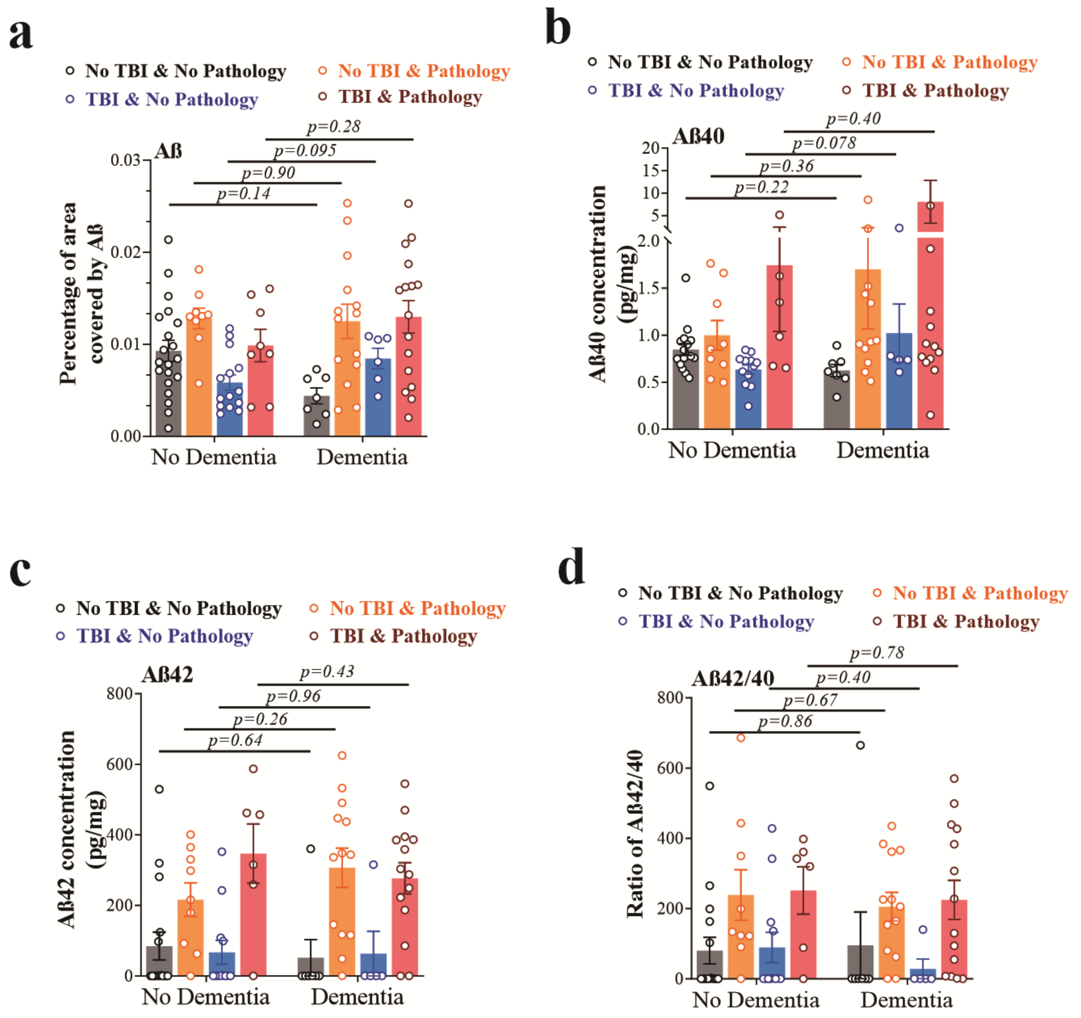

Figure S2. Aß related indexes showed no significant differences between different groups. a. Levels of percentage of area covered by Aß detected by IHC among different groups. **b.** Levels of Aß40 detected by Luminex assays among different groups. **c.** Levels of Aß42 detected by Luminex assays among different groups. **d.** Ratio of Aß42/40 detected by Luminex assays among different groups. The data are presented as the means ± SEM. Two-way ANOVA with Sidak's multiple comparisons test (**a-d**) was used. P values are indicated on the graphs.

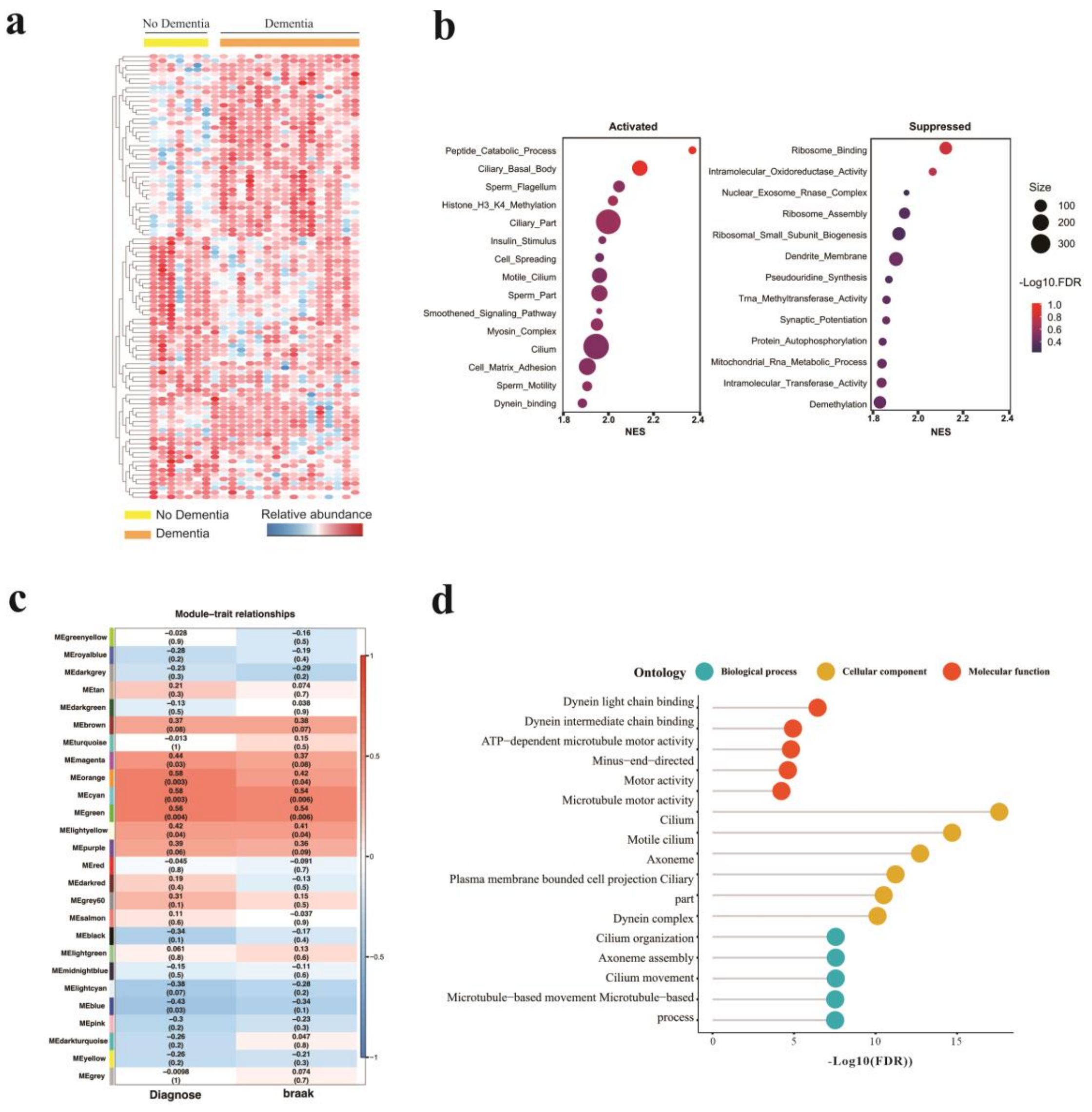

Figure S3. Cilium and dynein pathways were related to the pathology of post-traumatic brain injury neurodegeneration. a. Heatmap analysis of the top 200 different expression genes between the dementia group and non-demented group, which was calculated by limma package in R as described in methods. **b.** The top 10 significantly active and suppressed pathways in dementia patients, based on gene set enrichment analysis (GSEA). **c.** The correlation coefficients and p-values of gene modules with dementia diagnosis and Braak stage, and colors representing the magnitude of the correlation coefficients. **d.** The Go pathway of the gene model most relevant to dementia.

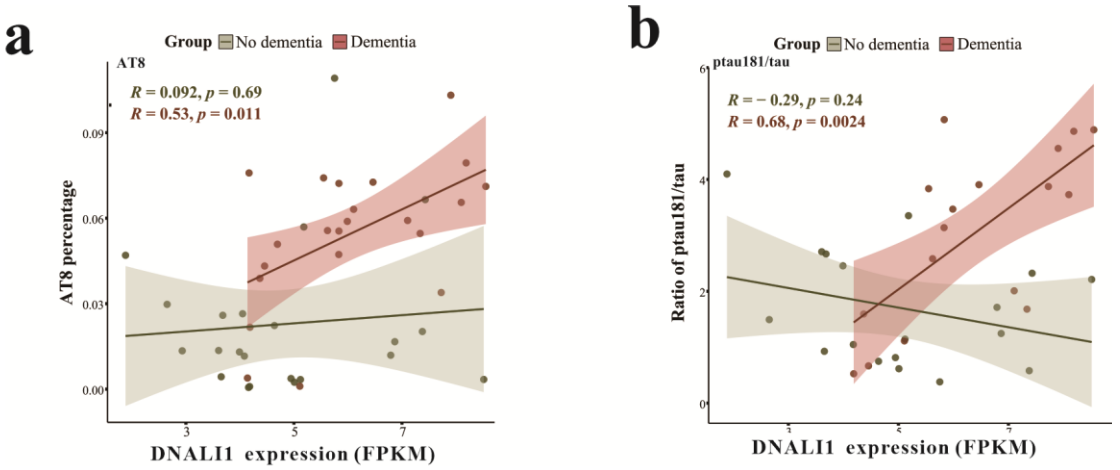

Figure S4. *DNALI1* expression was significantly correlated with AT8, or the ratio of ptau181/tau. a. The correlation between *DNALI1* expression fragments per kilobase per million (FPKM) and the percentage of area covered by AT8 for dementia and non-dementia groups. **b.** The correlation between *DNALI1* expression (FPKM) and the ratio of ptau181/tau for dementia and non-dementia groups. Pearson Correlation was used. P values are indicated on the graphs.

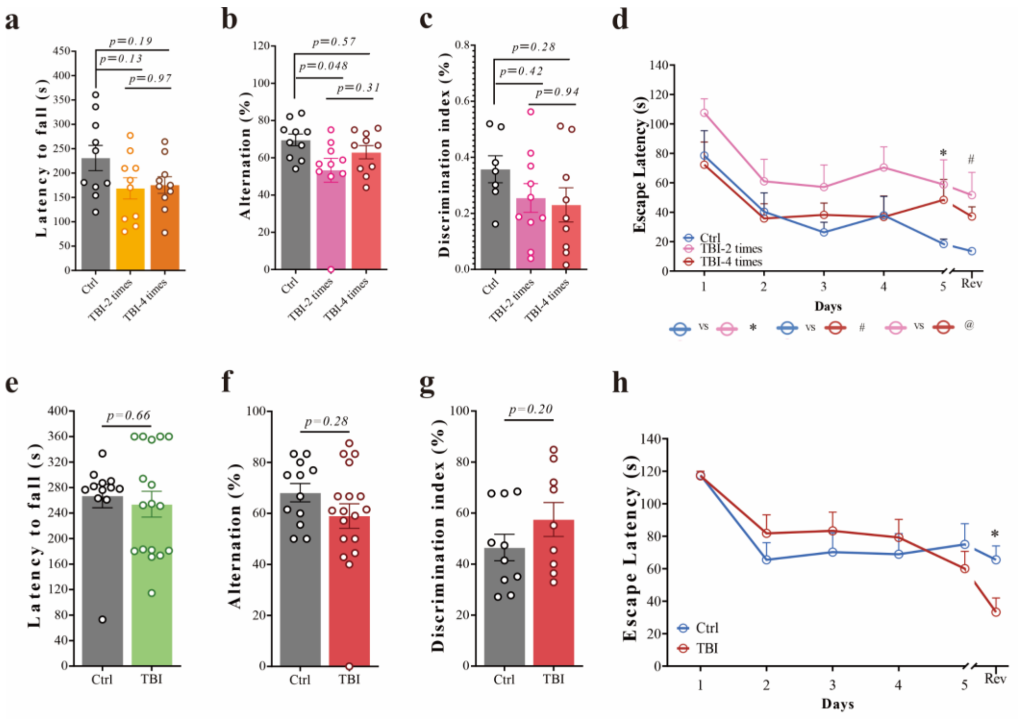

Figure S5. Two mild replicate hits and one month after hit is optimal for experiments. a. The performances on the rotarod test for different times of repetitive impact groups were analyzed one month after the brain injury (Ctrl, n = 10; TBI-2 times, n = 10; TBI-4 times, n = 10). **b.** The performances on the Y-Maze spontaneous alternation test for different times of repetitive impact groups were analyzed one month after the brain injury (Ctrl, n = 10; TBI-2 times, n = 10; TBI-4 times, n = 10). **c.** The performances on the Novel object recognition test for different times of repetitive impact groups were analyzed one month after the brain injury (Ctrl, n = 10; TBI-2 times, n = 10; TBI-4 times, n = 10). **d.** The performances for different times of repetitive impact groups on the Morris water maze test were analyzed one month after the brain injury (Ctrl, n = 10; TBI-2 times, n = 10; TBI-4 times, n = 10). **e.** The performance on the rotarod test was analyzed six months after the 2-times-hit brain injury (Ctrl, n = 12; TBI, n = 12). **f.** The performance on the Y-Maze spontaneous alternation test was analyzed six months after the 2-times-hit brain injury (Ctrl, n = 12; TBI, n = 12). **g.** The performance on the Novel object recognition test was analyzed six months after the 2-times-hit brain injury (Ctrl, n = 12; TBI, n = 12). **h.** The performance on the Morris water maze test was analyzed six months after the 2-times-hit brain injury (Ctrl, n = 12; TBI, n = 12). The data are presented as the means ± SEM. One-way ANOVA with Tukey's multiple comparisons test (**a-d**) and T-test (**e-h**) were used. P values are indicated on the graphs, * & #: $p < 0.05$.

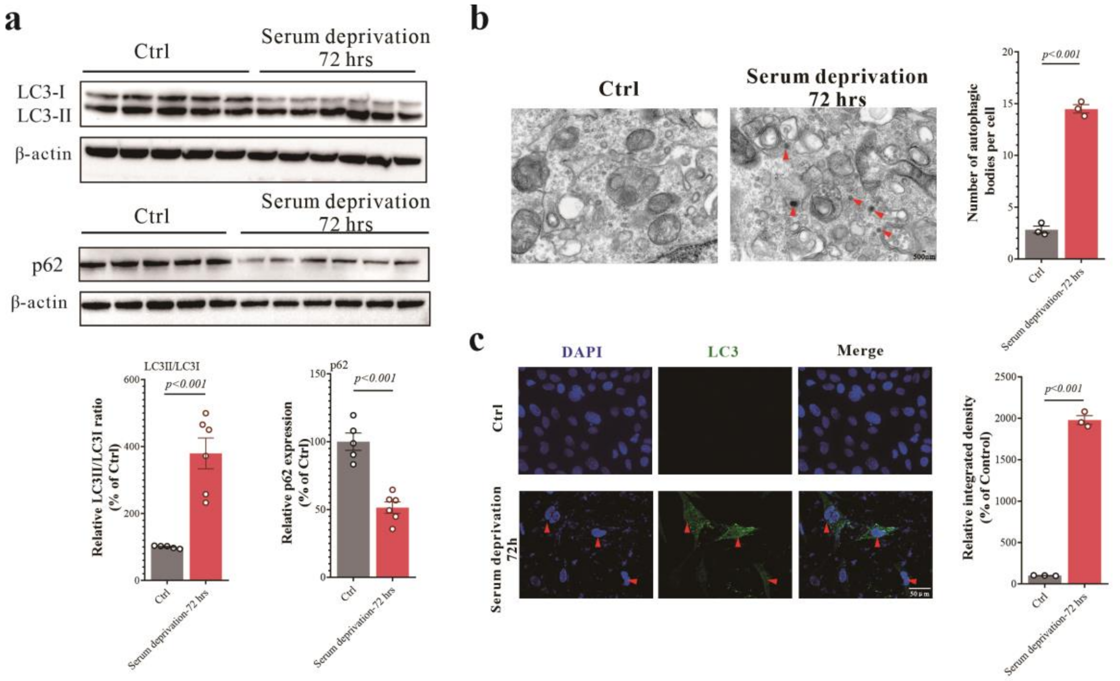

Figure S6. Autophagy level was activated after TBI. a. Western blot analysis of LC3 and p62 after 72h serum depletion (Ctrl, n = 5; TBI, n = 6). The data are normalized to β-actin and expressed relative to the control. **b.** Transmission electron microscopy images and quantification of the number of autophagic bodies for control and serum depletion cells, red arrowheads indicate autophagic vacuoles. Scale bars, 5000 nm, as indicated. **c.** Immunofluorescent colabeling of Lc3 (green) and nuclear (blue) and corresponding statistical results after 72h serum depletion. n = 3. Scale bar, 50 μm, as indicated. The data are presented as the means ± SEM. T-test (**a-c**) was used. P values are indicated on the graphs.

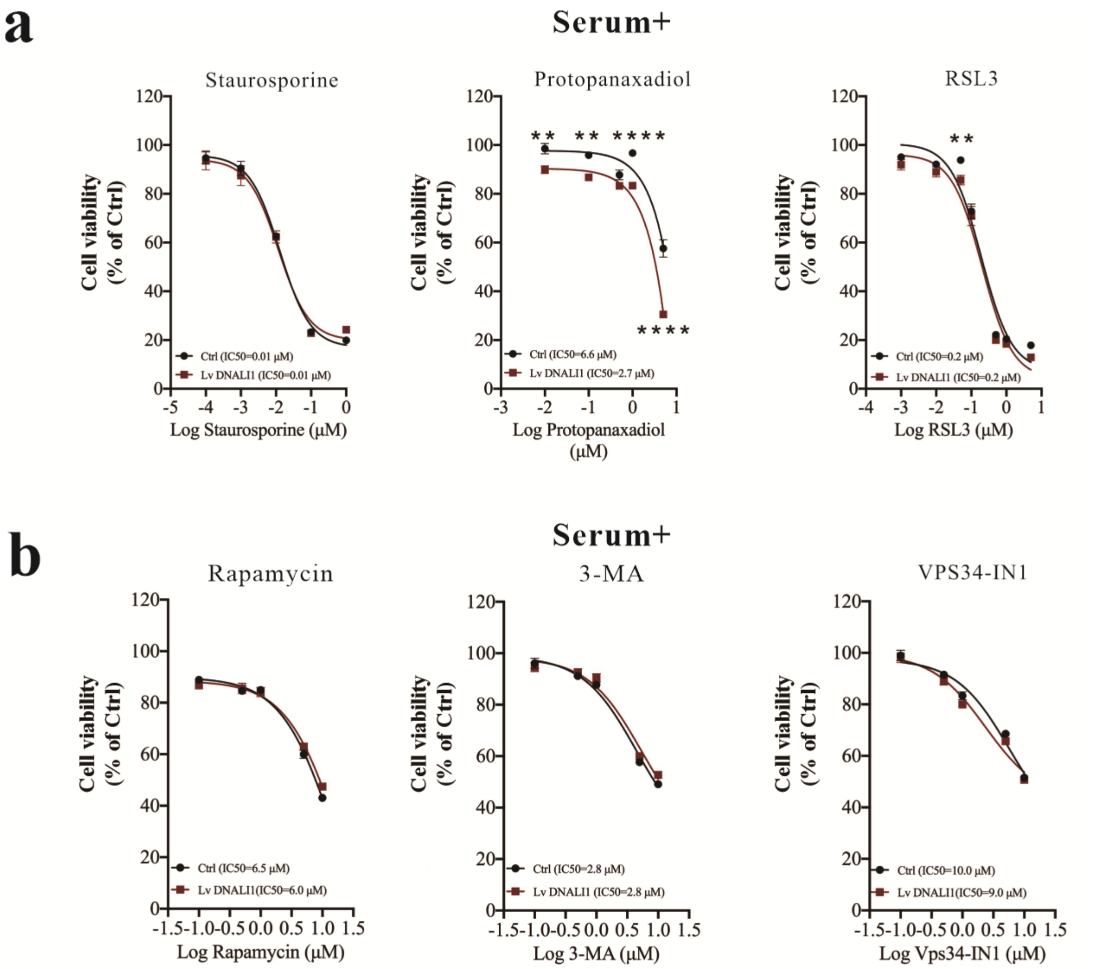

Figure S7. DNALI1 overexpression did not affect the cell susceptibilities to cell death and autophagy regulators. a. Cell viability of normal and DNALI1 overexpression cells after staurosporine, protopanaxadiol, and RSL3 treatment under serum normal status, n = 6 wells from one representative of 3 independent experiments. **b.** Cell viability of normal and DNALI1 overexpression cells after Rapamycin, 3-MA, or VPS34-IN1 treatment under serum normal status, n = 6 wells from one representative of 3 independent experiments**.** The data are presented as the means ± SEM. Two-way ANOVA with Sidak's multiple comparisons test (**a-b**) was used. P values are indicated on the graphs.

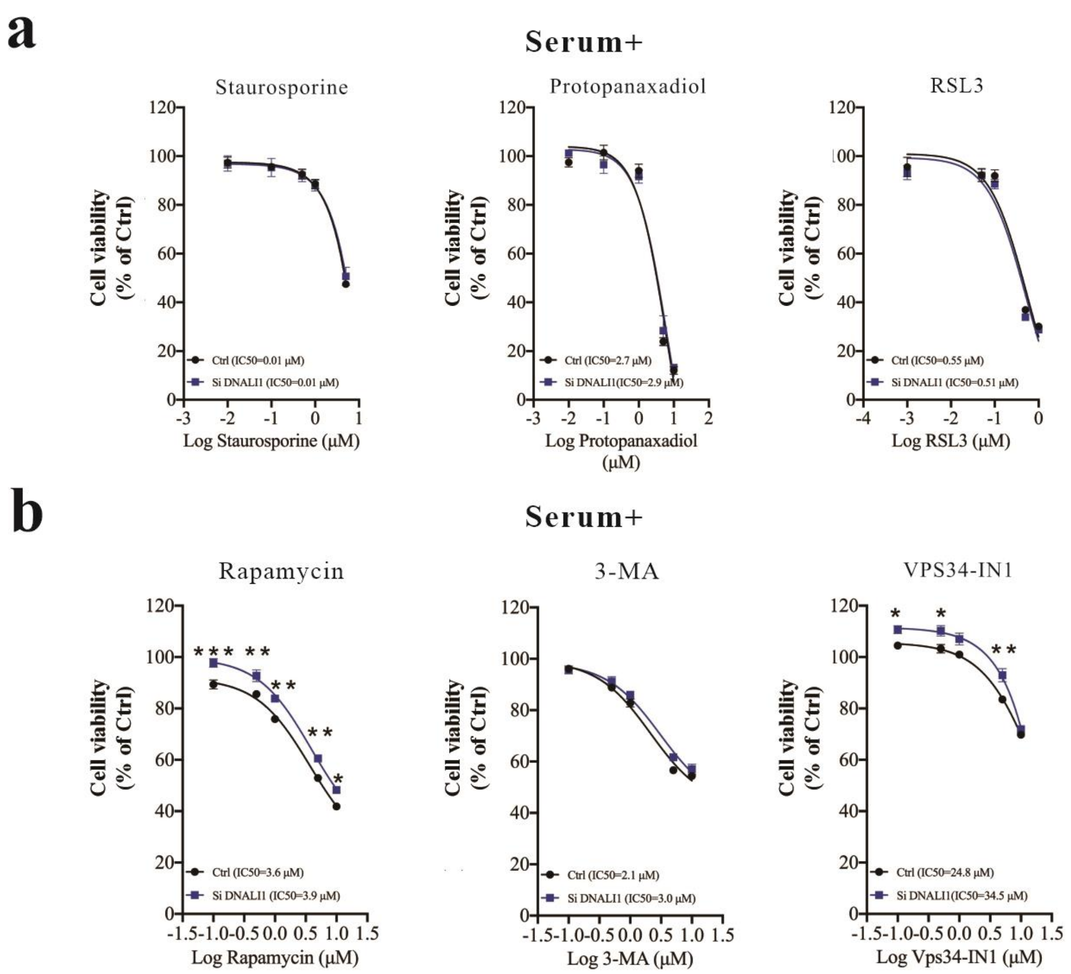

Figure S8. DNALI1 inhibition did not affect the cell susceptibilities to cell death regulators significantly. a. Cell viability of normal and DNALI1 inhibition cells after staurosporine, protopanaxadiol and RSL3 treatment under serum normal status, n = 6 wells from one representative of 3 independent experiments. **b.** Cell viability of normal and DNALI1 inhibition cells after Rapamycin, 3-MA, or VPS34-IN1 treatment under serum normal status, n = 6 wells from one representative of 3 independent experiments**.** The data are presented as the means ± SEM. Two-way ANOVA with Sidak's multiple comparisons test (**a-b**) was used. P values are indicated on the graphs.

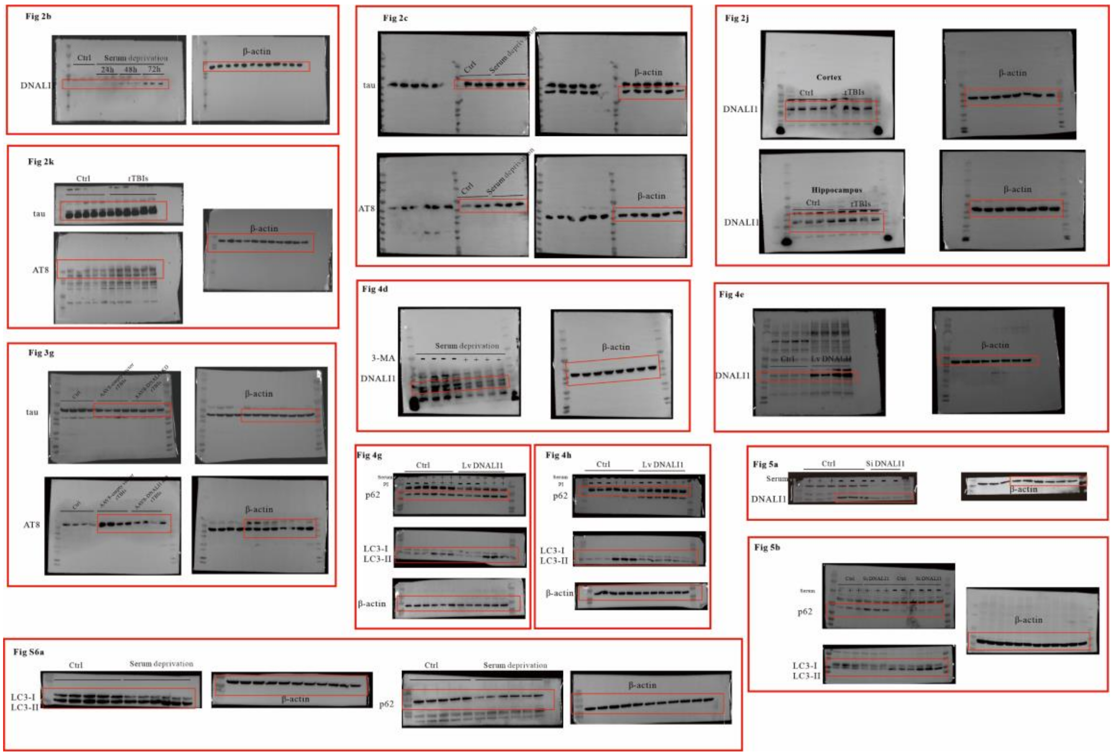

Figure S9. All uncropped images of the western blots in the study.

Table S1. Demographic Characteristics of Aging, Dementia and Traumatic Brain Injury Study.

	Control (n=57)	Dementia (n=50)	P value
Age, mean (SD) years	88.44 (6.71)	90.18 (6.46)	0.176[a]
Education, mean (SD) years	14.68 (3.22)	13.54 (3.16)	0.067[a]
Braak stage	2.82 (1.49)	4.1 (1.74)	< 0.001
CERAD score	1.23 (0.91)	1.76 (1.20)	0.01[a]
Presence of ApoE4 allele	7	13	0.085[b]
Ever TBI with Loss of Consciousness	27	26	0.70[b]
Longest duration of loss of consciousness			
< 10 sec	11	8	—
10 sec - 1 min	0	2	—
1-2 min	3	2	—
3-5 min	2	1	—
6-9 min	1	0	—
10 min - 1 h	4	4	—
> 1 h	4	4	—

Total, n = 107. Data are presented as mean (SD) or n. TBI = Traumatic brain injury; CERAD = Consortium to Establish a Registry for Alzheimer's Disease.

a: P-values from Student's T-Test test.

b: P-values from the chi-square test.

Table S2. Short interfering RNA sequences.

Target		Sequences
DNALI1	Forward	CCCAACMGGAAGGCAUUAUTT
	Reverse	AUAAMGCCUUCCAGUMGGGTT

Table S3. DNA sequences bound by sgRNAs.

sgRNA		Target sequences
DNALI1-mouse-sp	Forward	TGGCTGTGGGCTTGTACTAG
	Reverse	CTAGTACAAGCCCACAGCCA
Empty	Forward	GCACTACCAGAGCTAACTCA
	Reverse	TGAGTTAGCTCTGGTAGTGC
SiRNA- *DNALI1*	Forward	CCCAACMGGAAGGCAUUAUTT
	Reverse	AUAAMGCCUUCCAGUMGGGTT
